# Congestive Heart Failure Hospitalizations and Cannabis Use Disorder (2010–2014): National Trends and Outcomes

**DOI:** 10.7759/cureus.8958

**Published:** 2020-07-02

**Authors:** Temitope Ajibawo, Uvie Ajibawo-Aganbi, Farla Jean-Louis, Rikinkumar S Patel

**Affiliations:** 1 Internal Medicine, Brookdale University Hospital Medical Center, New York City, USA; 2 Health Sciences, Essen Health Care, New York City, USA; 3 Psychiatry, Griffin Memorial Hospital, Norman, USA

**Keywords:** congestive heart failure, cannabis use disorder, marijuana, trends, mortality, length of stay

## Abstract

Background and objectives: Prior studies have suggested that cannabis use is an independent risk factor for heart failure. With increasing recreational use of cannabis and decriminalization policies, cannabis use is expected to add to the burden of heart failure, but there is still limited data. Therefore, we utilized the Nationwide Inpatient Sample (NIS) database (2010-2014) to study the national trends and outcomes among cannabis users admitted for congestive heart failure (CHF).

Methods: We queried the NIS database and identified CHF as the primary diagnosis with a co-diagnosis of cannabis use disorder (CUD). Trends were analyzed with the linear-by-linear association.

Results: Total CHF admissions (N = 4,596,024) with comorbid CUD (N = 23,358 (0.5%)) were identified. An increasing prevalence trend from 0.4% to 0.7% (P= 0.001) was seen. CUD patients had a mean age of 49.78 years, 79% were males, 55.4% were African Americans, and 73.6% earn ≤ 50th percentile median household income of the patient’s ZIP code. Inpatient deaths (1.1% vs. 3.1%) were lower (P<0.001), and mean length of stay (LOS) was shorter among cannabis users compared to non-users (P=0.001). The mean LOS and total hospitalization costs demonstrated an increasing trend (P_trend_ = 0.001 and P_trend_ < 0.001) respectively. Alcohol abuse and depression were more prevalent among CUD compared to non-CUD patients.

Conclusion: CUD was associated with reduced inpatient deaths, but the prevalence of CUD and hospital charges is on the rise in the CHF inpatient population in addition to shorter mean LOS. Notwithstanding, these above findings prompt further research into its underlying mechanisms along with a probable causal relationship between cannabis and heart failure.

## Introduction

The past decade has witnessed unprecedented legalization and decriminalization of cannabis use across many states in the United States [1]. However, cannabis still maintains its status as a strictly prohibited drug at the federal level [1]. Marijuana is currently the most commonly used recreational drug in the United States, with >22 million users per month [2]. As of December 2018, marijuana was legal for medicinal use in thirty-three states and the District of Columbia (DC), while ten states and DC have enacted policies enabling recreational marijuana use [3].

Heart failure constitutes a significant public health issue, and its estimated prevalence is >5.7 million in the United States (US) [4]. Heart failure also constitutes a huge strain on the health care system, responsible for health care costs greater than $39 billion annually in the US [4]. The lifetime risk of developing heart failure is estimated to be 20% [4]. Thus, the consensus from a few published case reports and studies is that cannabis use may be associated with the development of left ventricular dysfunction and heart failure [5-8]

However, there is a dearth of systematic studies evaluating outcomes and trends of cannabis use disorder (CUD) in acute heart failure.

Based on limited data on comorbid CUD in heart failure patients, our study used Nationwide Inpatient Sample (NIS) data to determine the trends of demographic factors, in-hospital outcomes, and comorbidities for congestive heart failure (CHF) hospitalizations and cannabis use. Next, we compared in-hospital outcomes among CUD vs. non-CUD patients admitted for CHF

## Materials and methods

Data source

We used the NIS data in our study and diagnostic information was obtained using the International Classification of Diseases, Ninth Revision, Clinical Modification (ICD-9-CM) codes, and clinical classification (CCS) codes [9].

Inclusion criteria

We identified 4,596,024 patients with a primary discharge diagnosis of CHF using ICD-9-CM codes of 398.91, 402.01, 402.11, 402.91, 404.01, 404.03, 404.11, 404.13, 404.91, 404.93, 428.0-428.9 and 23,358 patients with co-morbid CUD using ICD-9-CM codes 304.30-304.32, and 305.2.

Variables of interest

To study the demographic trends in patients admitted for CHF with CUD from 2010-2014, we included demographic factors such as age, race, gender, household income, and primary payer status [10]. We compared the baseline demographics and in-hospital outcomes-length of stay (LOS), in-hospital mortality, and total hospital costs) between CUD and non-CUD patients admitted for CHF.

Comorbidities were considered coexisting clinical conditions to CHF. For assessment of comorbidities, the Agency for Healthcare Research and Quality (AHRQ) comorbidity software was used to create binary variables [11], then ICD-9 CM diagnosis codes were used to identify comorbidities. The ICD-9 CM codes used to determine the comorbid conditions are available in the appendices. We also applied discharge weights (DISCWT) to obtain a nationwide representative sample population [10].

Statistical analyses

Statistical analysis was done by SPSS version 25 (IBM, Armonk, NY, US). We used the independent sample t-test and analysis of variance (ANOVA) for measuring continuous data. Pearson’s chi-square test was used for categorical data. Trends were analyzed with the linear-by-linear association tests. A p-value ≤ 0.05 was used to define the statistical significance of the tests. The NIS database did not contain any personally identifiable information. Therefore, an institutional review board (IRB) approval was not needed for this study.

## Results

Demographics, inpatient outcomes and trends

The total number of admissions identified with a primary diagnosis of CHF over the five years was N = 4,596,024, and 0.5% (N = 23,358) of these patients had a co-diagnosis of CUD as shown in Table 1 below.

**Table 1:** Baseline characteristics of CHF hospitalizations by comorbid CUD. P ≤ 0.05 indicates statistical significance. CHF: congestive heart failure; CUD: cannabis use disorder; USD: United States Dollars; N: number; SD: standard deviation.

	CHF, N = 4,596,024				
Variable	Non-CUD		CUD		P-Value
	N	%	N	%	
Total admissions	4,572,666	99.5	23,358	0.5	
Mean age at admission (SD), in years	72.77 (14.43)		49.78 (11.24)		<0.001
Sex					
Males	2,296,127	50.2	18,448	79.0	<0.001
Females	2,276,092	49.8	4910	21.0	
Race					
White	2,920,078	68.4	7111	31.9	<0.001
African Americans	831,467	19.5	12,362	55.4	
Hispanic	32,090	7.5	1803	8.1	
Other	197,758	4.6	1034	4.6	
Median household income, in percentile					
0–25th	1,481,175	33.1	11,666	51.8	<0.001
26th–50th	1,184,624	26.4	5157	22.9	
51st–75th	1,020,324	22.8	3906	17.4	
76th–100th	793,574	17.7	1781	7.9	
Primary payer					
Medicare	344,360	75.5	6505	27.9	<0.001
Medicaid	369,350	8.1	8950	38.5	
Private	523,995	11.5	2488	10.7	
Self-pay or uninsured	147,146	3.2	4164	17.9	
Others	79,127	1.7	1169	5.0	
Comorbidities					
Alcohol abuse	126,464	2.8	6075	26.0	<0.001
Depression	4,555,384	10.0	2500	10.7	<0.001
Diabetes, without complications	1,574,996	34.4	5704	24.4	<0.001
Diabetes, with complications	478,489	10.5	3700	7.7	<0.001
Hypertension	3,436,096	75.1	17,276	74.0	<0.001
Metastatic cancer	45,995	1.0	89	0.4	<0.001
Renal failure	188,1371	41.1	7153	30.6	<0.001
Severity of illness					
Nil	221	<0.1	0	0.0	0.001
Minor	324,608	7.1	2263	9.7	
Moderate	1,680,851	36.8	9154	39.2	
Major	2,566,986	56.1	11,941	51.1	
Other hospital outcomes					
Mean length of stay (SD), in days	5.19 (5.82)		4.62 (4.98)		0.001
Mean total charges in (USD)	40,730		41,642		0.08
In-hospital mortality	140,878	3.1	249	1.1	<0.001

The proportion of total CUD admissions had an increasing trend from 0.4% (N = 3588) in 2010 to 0.7% (N = 6365) in 2014, which represents a 77.4% (P_trend_ < 0.001) increase over the five years as shown in Table 2 below.

**Table 2:** Trends of demographics and hospital outcomes of CUD in CHF inpatients. P ≤ 0.05 indicates statistical significance. CHF: congestive heart failure; CUD: cannabis use disorder; USD: United States Dollars; SD: standard deviation.

Variables	2010	2011	2012	2013	2014	Overall Total	P_trend_	Trend Direction
No Of CUD admissions	3588	3970	4305	5130	6365	23,358		
CUD admissions prevalence (%)	0.4	0.4	0.5	0.6	0.7	0.5	<0.001	Increasing
Age at admission								
Mean age (SD) (in years)	48.08 (11.41)	48.93 (11.22)	50.11 (11.22)	50.25 (10.81)	50.65 (11.38)	49.78 (11.24)	<0.001	Increasing
Gender (%)								
Males	81.7	78.9	81.2	78.9	76.0	79	0.001	Decreasing
Females	18.3	21.1	18.8	21.1	24.0	21.0	0.001	Increasing
Race (%)								
Whites	25.7	33.7	27.8	33.9	35.3	31.9	0.466	Increasing
African Americans	64.1	52.8	57.8	53.6	52.1	55.4	0.466	Decreasing
Hispanics	7.4	9.7	8.3	7.8	7.6	8.1	0.466	Increasing
Others	2.8	3.8	6.2	4.7	5.0	4.6	0.466	Increasing
Income level (%)								
0–25th percentile	54.3	49.7	53.2	48.5	53.5	51.8	0.851	Variable
26th–50th percentile	22.7	21.1	21.7	25.4	23.0	22.9	0.851	Variable
51st–75th percentile	16.8	19.6	17.8	17.4	15.9	17.4	0.851	Variable
76th–100th percentile	6.2	9.5	7.3	8.8	7.6	7.9	0.851	Increasing
Insurance (%)								
Medicare	26.3	27.2	28.3	27.9	29.1	27.9	<0.001	Increasing
Medicaid	34.5	35.6	36.8	36.5	45.1	38.4	<0.001	Increasing
Private	13.2	11.5	10.0	10.4	9.4	10.7	<0.001	Decreasing
Self-pay	20.2	19.2	18.6	19.5	14.0	17.9	<0.001	Decreasing
Other	5.7	6.4	6.3	5.7	2.4	5.0	<0.001	Variable
Severity of Illness (%)								
Minor	8.9	8.8	10.9	11.4	8.5	9.7	0.946	Variable
Moderate	39.4	37.5	41.8	39.3	38.3	39.2	0.946	Variable
Major	51.7	53.6	47.3	49.3	53.3	51.1	0.946	Variable
Other hospital outcomes								
In-hospital mortality (%)	0.7	0.8	1.5	1.2	1.0	1.1	0.137	Increasing
Mean total charges in USD	34,776	44,100	41,729	42,451	43,292	41,642	<0.001	Increasing
Mean length of stay (SD) (in days)	4.21 (3.58)	4.61 (4.39)	4.72 (5.30)	4.76 (5.95)	4.66 (4.91)	4.62 (4.98)	0.001	Increasing

The mean age of CUD patients increased significantly from 48.08 ± 11.41 years in 2010 to 49.78 ± 11.24 years (P_trend_ < 0.001). Males (79%) constituted most of the cannabis users, but there was a significant declining trend of CUD prevalence from 81.7% in 2010 to 76.0% in 2014 (P_trend_ = 0.001). Conversely, CUD prevalence among females increased from 18.3% to 21.0% in the five years (P_trend_ = 0.001). More than half of cannabis users were African Americans, followed by Caucasians (31.9%), Hispanics (8.1%), and others (4.6%). CUD prevalence among Whites and Hispanics followed an increasing trend but followed a decreasing trend among African Americans. All race groups showed a non-statistically significant trend (P_trend_ = 0.466).

Regarding median household income in percentiles, CUD prevalence among patients below the 25th percentile, 26th to 50th percentile, and 51st to 75th percentile showed a variable trend, while CUD prevalence in the highest percentile demonstrated an increasing trend from 2010 to 2014 (P_trend_ = 0.851). Patients below the 25th percentile represented about half of the CUD admissions.

In terms of insurance status, Medicaid was the primary insurance for 38.4% of the total CUD population, while Medicare was the primary payer for 27.9%. Both Medicaid and Medicare payments had an increasing trend (P_trend_ < 0.001). Private insurance and self-pay mode of payment followed statistically significant down-trend payments from 13.2% to 9.4% and 20.2% to 14% over the five years (P_trend_ < 0.001) respectively.

Cannabis users with major morbidity made up 51.1% of the total CUD population, and there was a non-statistically significant variable trend from January 2010 to December 2014 (P_trend_ = 0.946). In-hospital mortality (1.1% vs. 3.1%), was lower, and mean LOS in days were shorter in CUD patients compared to the non-CUD cohort (P < 0.001) and (P = 0.001) respectively. There was a non-statistical difference in total hospital charges between CUD and non-CUD patients (P = 0.08).

In-hospital mortality among CUD patients followed a non-statistically significant trend from 0.7% to 1.1% from 2010 to 2014 (P_trend _< 0.137), but there were a significant trend increase from 2010 to 2014 for both LOS in days (P_trend_ = 0.001) and total hospital charges (P_trend _< 0.001).

Comorbidities and trends

**Figure 1:**
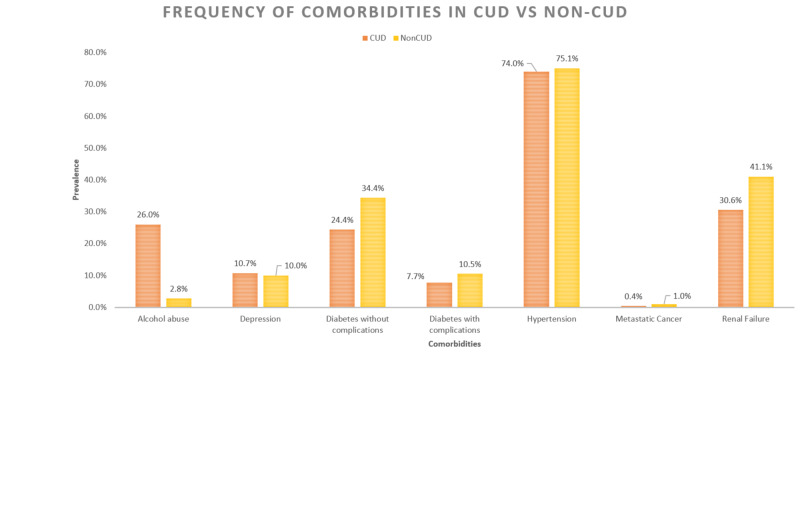
Frequency of comorbid conditions in CUD vs. non-CUD CHF inpatients. CHF: congestive heart failure; CUD: cannabis use disorder.

Comorbidities of diabetes, hypertension, renal failure, and metastatic cancer were less prevalent among cannabis users as shown in Figure 1 below.

At the same time, alcohol abuse and depression were more prevalent among CUD patients. Alcohol abuse showed a significant decreasing trend from 2010 to 2014 (P_trend_ < 0.001) as shown in Table 3 below

**Table 3:** Trends of comorbidities associated with CUD in CHF patients. p ≤ 0.05 indicates statistical significance. CHF: congestive heart failure; CUD: cannabis use disorder.

Comorbidities	2010	2011	2012	2013	2014	Total	P _trend_	Trend Direction
Alcohol abuse	27.1%	26.9%	27.1%	26.1%	24.0%	26.0%	<0.001	Decreasing
Depression	10.4%	11.4%	10.1%	10.3%	11.2%	10.7%	0.621	Variable
Diabetes without complications	22.1%	22.4%	26.5%	24.9%	25.2%	24.4%	<0.001	Increasing
Diabetes with complications	6.2%	8.1%	6.6%	8.0%	8.6%	7.7%	<0.001	Increasing
Hypertension	73.9%	73.4%	74.7%	73.2%	74.5%	74.0%	0.590	Variable
Metastatic Cancer	0.3%	0.4%	0.1%	0.8%	0.3%	0.4%	0.126	Variable
Renal failure	30.6%	30.6%	29.8%	30.4%	31.3%	30.6%	0.412	Stable

Conversely, diabetes, with and without complications significantly increased in a linear trend from 2010 to 2014 (P_trend_ < 0.001).

Comorbidities of hypertension (P_trend_ = 0.590), depression (P_trend_ = 0.621), and metastatic cancer (P_trend _= 0.126) showed a non-significant variable trend. Renal failure was the only comorbidity that showed a stable linear trend. 

## Discussion

This study considered demographic factors (age, race, gender, insurance type, household income), the severity of illness, in-hospital mortality, total hospital charges, and LOS in both cannabis users and non-users. Trends of coexisting comorbidities among CHF hospitalizations with CUD was also explored. We reported an overall prevalence of CUD among CHF inpatients as 0.5% and a significant trend of 77.4%. These findings are similar to a recent study by Charilaou et al. that reported an increase in cannabis abuse prevalence in patient populations from 0.5% to 1.3% from 2002 to 2011 [12]. Our observation may be partially explained by the legalization of medical and recreational cannabis in the many U.S. states [13,14], which consecutively led to an increase in the prevalence of cannabis users in the general population, and by extension increased the odds of observing them in admitted patients, including CHF hospitalizations [12].

In another study of cannabis use, Kalla et al. showed that cannabis use was an independent risk predictor of heart failure [8]. Evidence of this association between cannabis use and heart failure is backed by prior human and animal studies [15-18]. Three earlier studies showed that endocannabinoids acting on CB-1 receptors caused decreased myocardial contractility [15-17]. Su et al. also showed that the negative inotropy activity of CB2 agonists, independent of CB1 and CB2 receptors, causes decreased myocardial contractility [18].

Our study showed that cannabis users were predominantly males (79%) with a mean age of 49.8 years. These findings are consistent with findings by Wu and colleagues in a study of the effects of marijuana use on heart failure, which reported a mean age of 50.4 years and predominance of males (78.3%) among marijuana users compared to non-users [19].

Two notable findings of this study are the lower mortality rate (1.1% vs. 3.1%) and reduced LOS (4.62 vs. 5.12 days) among cannabis users compared to non-cannabis users. These results are similar to findings of a previous study that marijuana use was associated with reduced inpatient mortality (odds ratio (OR): 0.197 (0.046-0.083) p = 0.0142) and reduced LOS (4.2 ± 0.1 vs. 4.8 ± 0.2, p = 0.0038) in patients admitted for heart failure [19]. We hypothesized that the lower mortality among CUD patients admitted for heart failure is due to the relatively younger age of this cohort, which makes it possible that the baseline risk of dying in these patients was less than that of non-cannabis users. Another possible explanation for the lower mortality is reduced severity of illiness among cannabis users.

A possible explanation for the reduced mean LOS among cannabis users could be a reduced severity of CHF illness. Notwithstanding these findings require further investigation in the future. This study demonstrated a statistically significant increase in LOS among cannabis users from 2010 to 2014, which we believe caused an increase in hospital charges from ($34,776 to $43,292). Unlike our study which did not show any significant differences in hospital costs between CUD and non-CUD patients, a prior study by Wu et al. [19] demonstrated a significantly lower hospital cost among cannabis users vs. non-users. Although the reason for the above finding is largely speculative, this contrasting finding from previous observation is a significant addition to the body of literature.

Our study revealed essential differences in demographic factors between CUD and non-CUD patients admitted for CHF. Patients hospitalized for CHF with CUD were mostly younger men of African American origin and earned less than the 50th percentile household income. Like our study, it was reported by Nishimura et al. that patients hospitalized for heart failure who had a history of substance abuse were mostly males, and likely to be African American and younger [20].

Alcohol abuse (74% vs. 26%) had a higher prevalence among cannabis users compared to non-CUD. Previous studies of cannabis use in the inpatient population support this finding. Charilaou et al. explored comorbidities associated with cannabis use disorder in inpatients between 2002 to 2011 using the AHRQ comorbidity indicators and reported alcohol abuse as one of the primary comorbid conditions associated with cannabis use [12]. In the same study, approximately 33% of inpatient cannabis users were reported to have a history of alcohol abuse [12]. Similarly, another study that reported alcohol study surveys of marijuana and other illicit drugs revealed that most marijuana users were either binge drinkers or users of other substances [21]. Furthermore, Smit and Crespo studied the nutritional status of adult cannabis users and found out that cannabis users consume higher proportions of alcohol compared to non-users [22]. It is worth mentioning that alcohol abuse is associated with alcoholic cardiomyopathy [23], which is characterized by increased left ventricular mass, left ventricular dilation along with reduced and/or normal ventricular wall thickness [24]

Alcohol abuse as a comorbidity demonstrated a statistically significant decreasing trend from 2010 to 2014. A possible explanation for this phenomenon is the “substitution effect” of alcohol with cannabis use and other illicit drugs by cannabis users [25,26].

Our results showed a higher prevalence of depression among cannabis users vs. non-users (10.7% vs. 10%). Long term use of cannabis has been previously linked with depression [27]. In a prior study of heart failure patients with depression, 15% of these patients were found to have substance abuse problems [28].

This present study has a few limitations that should be considered when interpreting the results. Based on the administrative nature of the NIS database, there is a possibility of coding inaccuracies and classification errors. Secondly, due to the contentious legal and societal status of cannabis use, reporting of cannabis abuse is prone to underreporting bias. Based on the nature of the database, there was unavailability of patient-level information; therefore, adjustment for confounders could not be performed during analysis. Our study, being an observational study, could not assess a temporal relationship between cannabis use and CHF. The level of exposure to cannabis use could not be assessed because of the unavailability of the information in the database, so we were unable to comment on the quantity of cannabis.

Despite these limitations, the NIS database provides an invaluable population-based resource for evaluating national trends. Another strength of this study is obtained from the large sample size of the NIS data, which has enough power to determine differences between CUD and non-CUD CHF populations. Findings from our study also serve as hypotheses and a valuable reference for further research.

## Conclusions

Among CUD patients hospitalized for CHF, the trend for the total number of admissions, mean LOS and mean total charges increased significantly over the five-year period of study. In addition, CUD patients had less all-cause in-hospital deaths and shorter hospital stays compared to non-users. Moreover, cannabis users followed a specific demographic profile as they were mostly males, African Americans, and lower-income earners. Further in-depth research is warranted to explore a causal relationship between cannabis use and the development of heart failure

## Appendices

**Table 4:** ICD-9 CM codes for co-morbid conditions

Alcohol abuse	305.00–305.03, 303.00–303.93, 291.9, 291.89, 291.81,291.8, 291.5, 291.0–291.3
Depression	311, 309.1, 309.0, 301.12, 300.4.
Diabetes without complications	648.00–648.04, 250.00–250.33, 249.00–249.31.
Diabetes with complications	775.1, 250.40–250.93, 249.40–249.91.
Hypertension	642.70–642.94,642.10–642.24, 642.00–642.04, 437.2, 402.00–405.99, 401.0, 401.1, 401.9.
Metastatic cancer	199.0–199.2, 198.0–198.89, 197.0–197.8, 196.0–196.9
Renal failure	586, 585.9, 585.6, 585.5, 585.4, 585.3, 404.93, 404.92, 404.13, 404.12, 404.03, 403.01, 404.02, 403.91, V56.8, V56.0-V56.32, V45.12, V45.1, V42.0.
